# Immobilization induced osteopenia is strain specific in mice

**DOI:** 10.1016/j.bonr.2015.04.001

**Published:** 2015-04-17

**Authors:** Andreas Lodberg, Jens Bay Vegger, Michael Vinkel Jensen, Christian Mirian Larsen, Jesper Skovhus Thomsen, Annemarie Brüel

**Affiliations:** Department of Biomedicine, Aarhus University, Aarhus, Denmark

**Keywords:** Immobilization, Osteopenia, Botox systemic effects, MicroCT, Biomechanics, Histomorphometry

## Abstract

Immobilization causes rapid and massive bone loss. By comparing Botulinum Toxin A (BTX)-induced bone loss in mouse strains with different genetic backgrounds we investigated whether the genetic background had an influence on the severity of the osteopenia. Secondly, we investigated whether BTX had systemic effects on bone. Female mice from four inbred mouse strains (BALB/cJ, C57BL/6 J, DBA/2 J, and C3H/HeN) were injected unilaterally with BTX (n = 10/group) or unilaterally with saline (n = 10/group). Mice were euthanized after 21 days, and the bone properties evaluated using μCT, DXA, bone histomorphometry, and mechanical testing. BTX resulted in substantially lower trabecular bone volume fraction (BV/TV) and trabecular thickness in all mouse strains. The deterioration of BV/TV was significantly greater in C57BL/6 J (− 57%) and DBA/2 J (− 60%) than in BALB/cJ (− 45%) and C3H/HeN (− 34%) mice. The loss of femoral neck fracture strength was significantly greater in C57BL/6 J (− 47%) and DBA/2 J (− 45%) than in C3H (− 25%) mice and likewise the loss of mid-femoral fracture strength was greater in C57BL/6 J (− 17%), DBA/2 J (− 12%), and BALB/cJ (− 9%) than in C3H/HeN (− 1%) mice, which were unaffected. Using high resolution μCT we found no evidence of a systemic effect on any of the microstructural parameters of the contralateral limb. Likewise, there was no evidence of a systemic effect on the bone strength in any mouse strain. We did, however, find a small systemic effect on aBMD in DBA/2 J and C3H/HeN mice. The present study shows that BTX-induced immobilization causes the greatest loss of cortical and trabecular bone in C57BL/6 J and DBA/2 J mice. A smaller loss of bone microstructure and fracture strength was seen in BALB/cJ mice, while the bone microstructure and fracture strength of C3H/HeN mice were markedly less affected. This indicates that BTX-induced loss of bone is mouse strain dependent. We found only minimal systemic effects of BTX.

## Highlights

• Botulinum Toxin A (Botox) causes only minimal systemic effects in mice.
• The degree of immobilization induced osteopenia is highly strain specific in mice.
• The greatest degree of bone loss was observed with C57BL/6 J and DBA/2 J mice followed by BALB/cJ mice after Botox-injection.
• C3H/HeN mice had the smallest bone loss following Botox-injection.

## 1 Introduction

Immobilized patients, whether due to upper or lower motor neuron damage, muscular dystrophies, or severe backaches, are prone to develop osteopenia and osteoporosis in the affected limbs (Jiang et al., 2006, Minaire et al., 1974, Leblanc et al., 1990, Rittweger et al., 2010, Thomsen et al., 2005a, Hansson et al., 1975, Krølner and Toft, 1983). For example, Hansson et al. found a loss of spinal bone mineral content of 2% per week in patients undergoing bed rest due to scoliosis surgery (Hansson et al., 1975), while Krølner and Toft showed that patients recumbent due to therapeutic bed rest had a loss of spinal bone density of 1% per week (Krølner and Toft, 1983). However, immobilizing an otherwise healthy human population raises a number of ethical questions, and problems related to tissue extraction and analyses. Instead, a range of animal models are available for the investigation of bone loss following skeletal unloading: Neurectomy (Kodama et al., 1999, Zeng et al., 1996, Weinreb et al., 1989, Sakai et al., 1999, Sakai et al., 2005), spinal cord injury (McManus and Grill, 2011, Ding et al., 2011), tail suspension (Morey, 1979), plaster casting (Rantakokko et al., 1999, Orimo et al., 1971), elastic bandaging (Akamine et al., 1992, Lindgren, 1976), and Botulinum Toxin A (BTX)-induced immobilization (Warner et al., 2006a). Here, we focus on the BTX model, which besides requiring little setup and being minimally invasive has been shown to have skeletal effects over and above any effect it has in altering gravitational loading, thereby suggesting a direct effect of muscle on bone (Warden et al., 2013).

After the injection of BTX, the ensuing muscle paralysis is based on the toxin's ability to cleave the synaptosome-associated protein 25 (SNAP-25) in the presynaptic membranes of the neuromuscular junction. This prevents vesicles containing acetylcholine from docking at the presynaptic membranes, and results in blockade of vesicle fusion and the subsequent release of acetylcholine into the synaptic cleft (Lin and Scheller, 2000, Hambleton, 1992, Dressler and Adib, 2005, Simpson, 1981). Unlike invasive methods of immobilization, like neurectomy and spinal cord injury, the BTX model preserves the sensory nerve function of the animals as only the efferent motor pathways become affected.

We have previously shown losses of bone density, microstructure, and strength in rats following BTX injection (Vegger et al., 2014, Thomsen et al., 2012, Brüel et al., 2013, Grubbe et al., 2014), and these findings are similar to what has been shown in mice (Warner et al., 2006a, Warden et al., 2013, Ellman et al., 2014, Manske et al., 2010a, Aliprantis et al., 2012, Grimston et al., 2007, Ausk et al., 2013, Manske et al., 2010b, Manske et al., 2012, Poliachik et al., 2010, Warner et al., 2006b). Furthermore, it has been demonstrated that the bone deterioration following BTX-induced muscle paralysis is paralleled by an increase in osteoclast activity and osteoclastogenesis, and these two factors are believed to play key roles for the rapidly occurring bone loss in this model of immobilization (Aliprantis et al., 2012, Warner et al., 2006b).

Mice are increasingly being used as experimental animals in bone research. Interestingly, skeletal features such as bone density, strength, and microarchitecture have been shown to differ between a variety of mouse strains (Jiao et al., 2010, Beamer et al., 1996, Sabsovich et al., 2008, Wergedal et al., 2005). Moreover, the response to skeletal challenges such as ovariectomy (OVX) has been shown to vary in different mouse strains. Hence, Bouxsein et al. showed a great mouse strain related variation in the bone loss following OVX in four strains of mice (Bouxsein et al., 2005). Studies of tail suspended mice have indicated that the response to skeletal unloading is also dependent on the mouse strain studied (Shahnazari et al., 2012, Amblard et al., 2003). When conducting experiments involving disuse in mice caution should be exercised when selecting the mouse strain for the study as the different mouse strains respond very differently to disuse. Hence, it is necessary to know how various mouse strains respond to BTX-induced bone loss.

Recently, the possibility of a systemic effect in BTX treated mice has attracted attention. Reports have been ranging from a single study showing a massive systemic impact (Ellman et al., 2014) to other studies presenting only negligible systemic effects of BTX (Manske et al., 2010a, Manske et al., 2010b, Poliachik et al., 2010).

Therefore, the main objective of the present study was to establish whether four commonly used mouse strains would respond differently when immobilized by BTX. In addition, we performed a comprehensive investigation of the possible systemic effect of BTX.

## 2 Materials & methods

### 2.1 Animals

Eighty 16-week-old female BALB/cJ (BALB/c) and C57BL/6 J (B6) mice (Taconic Farms, Denmark), and DBA/2 J (DBA) and C3H/HeN (C3H) mice (Harlan Laboratories, Holland) were investigated. The mice were randomized into one control (n = 10) and one experimental group (n = 10) per mouse strain and housed at 22 °C with a 12/12 h light/dark cycle with free ambulation and standard mice chow and water ad libitum.

On day 0 the mice were anesthetized with 4% isoflurane (Isofluran; Baxter, Deerfield, IL) by inhalation and injected IM with either saline or Botulinum Toxin A (BTX) (Botox, Allergan, Irvine, CA). The BTX dosage was 2 U/100 g body weight and was injected into the quadriceps musculature and the calf muscles. The contralateral (non-injected) hind limbs served as internal controls. During the study no adverse effects of the BTX injections were observed, and there were no visible signs of the immobilized animals not thriving.

The gait ability of the BTX-injected animals was assessed (as described by Warner et al., 2006a) in a pilot study of 10 BALB/c mice on days 0, 1, and 2, and thereafter twice a week.

All mice received SC injections with alizarin (Sigma-Aldrich, St. Louis, MO, 20 mg/kg) on day − 5, calcein (Sigma-Aldrich, St. Louis, MO, 15 mg/kg) on day 13, and tetracycline (Sigma-Aldrich, St. Louis, MO, 20 mg/kg) on day 17.

Body weights were obtained at days − 5, 0, 5, 12, and 21. The mice were euthanized on day 21. No mice died prematurely during the course of the study. All procedures complied with the guiding principles of the *Guide for the Care and Use of Laboratory Animals* and were approved by the Danish Animal Experiment Inspectorate.

### 2.2 Tissue extraction

Immediately after euthanasia the left and right hind limbs were removed. Both the left and right rectus femoris muscles were carefully isolated in a standardized manner and weighed using an electronic scale. The femora were stored in Ringer's solution at − 20 °C for the subsequent analyses. The tibiae were immersion-fixed in 0.1 M sodium phosphate-buffered 4% formaldehyde, pH 7.0, for 48 h and then transferred to 70% ethanol.

### 2.3 Dual-energy X-ray absorptiometry

The femora were thawed, cleaned of soft connective tissue, placed in a pDEXA scanner (Sabre XL; Norland Stratec, Pforzheim, Germany), and scanned using a pixel size of 0.1 × 0.1 mm^2^. The bone mineral content (BMC) and the area bone mineral density (aBMD) of the whole bone were determined with the software provided with the scanner. Quality assurance was performed by scans of the two solid-state phantoms provided with the scanner. The coefficient of variation (CV) of mice femoral aBMD is 2.4% in our laboratory.

### 2.4 Mechanical testing

The femoral length was determined using a digital caliper, and the midpoint was marked. The femora were placed in a testing jig constructed for three-point bending tests. The distance between the supporting rods was fixed at 7.15 mm. Load was applied at a constant deflection rate of 2 mm/min with a rod perpendicular to the midpoint of the femora in a materials testing machine (5566; Instron, High Wycombe, UK) until fracture.

The proximal femur obtained after the three-point bending test was placed in a device for standardized fixation (Mosekilde et al., 1999). The fixation device was then placed in the materials testing machine and load was applied to the top of the femoral head at a constant rate of 2 mm/min until fracture.

### 2.5 Micro-computed tomography (μCT)

The proximal tibial metaphyses were μCT-scanned (μCT 35; Scanco Medical, Brüttisellen, Switzerland) in high resolution mode (1000 projections/180°) with a spatial resolution of 3.5 × 3.5 × 3.5 μm^3^, an X-ray tube voltage of 55 kV_p_ and current of 145 μA, and an integration time of 800 ms.

A 1000-μm-high volume of interest (VOI) was delineated within the endocortical edges of the proximal tibial metaphysis starting 200 μm below the most distal part of growth plate in order to exclude the primary spongiosa. The 3D data sets were low-pass-filtered using a Gaussian filter (σ = 1.3, support = 2) and segmented with a fixed threshold filter of 476 mg HA/cm^3^ in accordance with the current guidelines (Bouxsein et al., 2010).

Determination of the trabecular bone microstructure was carried out using the software provided with the μCT scanner (version 6.0, Scanco Medical). The microstructural measures included bone volume per total volume (BV/TV), trabecular thickness (Tb.Th), trabecular separation (Tb.Sp), trabecular number (Tb.N), connectivity density (CD), structural model index (SMI), trabecular bone material density (ρ_trab_), and volumetric bone mineral density (vBMD). The computation of these structural measures was performed using the direct method as previously described in detail (Thomsen et al., 2005b).

Quality assurance was performed by weekly (density) and monthly (geometry) scans of the solid-state calibration phantom provided with the scanner.

### 2.6 Static bone histomorphometry

Approximately 200-μm-thick sections were sawed from the femoral mid-diaphysis with a diamond precision-parallel saw (Exakt Apparatebau, Norderstedt, Germany). The sections were sawed as closely as possible to the fracture site from the three-point bending test and perpendicular to the long axis of the bone. These unembedded sections were mounted on microscope slides and placed in a microscope (Eclipse 80i; Nikon, Tokyo, Japan) equipped with a motorized stage (H138E50; Prior, Cambridge, UK), a digital camera (DP72; Olympus, Tokyo, Japan), and connected to a PC with the newCAST stereology software (version 3.6.4.0; Visiopharm, Hørsholm, Denmark). Digital images were projected onto the computer monitor of the attached PC. At a magnification of × 290 a point grid was superimposed by the software and marrow area (Ma.Ar) and bone area (B.Ar) were estimated.

### 2.7 Dynamic bone histomorphometry

Cortical bone dynamic histomorphometry was performed using the mid-diaphyseal sections and the stereology system, which was also equipped with a fluorescence illuminator (LH-M100C-1; Nikon, Tokyo, Japan). A randomly rotated star shaped grid with 16 radiating arms was superimposed on the digital images. The center of the grid was placed in the center of the medullar cavity, so that the radiating lines of the grid randomly intersected the endosteal and periosteal perimeter as previously described (Oxlund and Andreassen, 2004). The number of grid intersections with either single labels or double labels at the endosteal or periosteal surface was counted at a magnification of × 1170. In the case of grid intersection with a double label, the distance between the labels was measured. Alz.S/BS, MS/BS, MAR, and BFR/BS of the cortical bone were determined.

After μCT, the proximal tibiae were embedded undecalcified in methyl methacrylate. Frontal 7-μm-thick sections were cut on a microtome (Jung RM2065; Leica Instruments, Nussloch, Germany) and either left unstained, stained for tartrate-resistant acid phosphatase (TRAP), or stained with Masson-Goldner trichrome (MGT).

Unstained sections were placed in the microscope and analyzed using newCAST. A 1000-μm-high region of interest (ROI) was delineated within the endocortical edges of the metaphysis starting 300 μm below the top of the growth plate, excluding primary spongiosa. The fields of view were sampled systematically random (Gundersen and Jensen, 1987), and a line grid with random orientation was superimposed on the fields of view. MS/BS, MAR, and BFR/BS of the trabecular bone were determined at a magnification of × 1170. The amount of osteoclast covered surfaces (Oc.S/BS) was estimated in a similar way using the sections stained for TRAP.

### 2.8 Bone adiposity of the proximal tibia

The adipose tissue volume relative to marrow volume (AV/MV) was estimated on the MGT stained sections using point counting in a ROI delineated from the bottom of the growth plate and 2 mm distally.

### 2.9 Statistics

To assess the systemic effect of BTX, the contralateral limbs from control mice were compared with contralateral limbs from BTX-treated mice (with the exception of μCT data where saline-injected limbs of control mice were used instead) using a Student's independent samples *t*-test. To demonstrate the magnitude of BTX-induced bone changes the absolute values obtained at the injected and contralateral limbs were compared with a Student's paired samples *t*-test. To evaluate the influence of mouse strain type on BTX-induced changes the ratios of BTX-injected to non-injected limbs from all four mouse strains were compared using a one-way analysis of variance with a Student-Newmann–Keuls post-hoc analysis. When normal distribution was not found, Wilcoxon Mann–Whitney *U* test, Wilcoxon Signed-Rank test, and Kruskal–Wallis one-way analysis of variance on ranks with a Student–Newmann–Keuls post-hoc analysis, respectively, were applied instead. Differences were regarded as statistically significant when *p* < 0.05. All data are expressed as mean ± SD or mean percent difference ± SD.

## 3 Results

### 3.1 Body weight and gait ability score

In the 5 days following BTX injection, the DBA and C3H strain lost weight significantly, whereas the BALB/c and B6 strains experienced only non-significant weight losses. At the end of the study, however, the body weights of BTX animals in both the BALB/c, DBA, and C3H, but not the B6 strain, were significantly lower (7–12%) than the body weights of their respective control animals.

The pilot study of 10 BALB/c mice showed that the gait ability score rapidly deteriorated, and reached its lowest (2.1 ± 1) at day 2, after which the mice slowly recuperated, and finished on a gait ability score of 6.6 ± 1 at day 21.

### 3.2 Rectus femoris muscle mass and femur length

In all mouse strains, a significant loss of rectus femoris muscle mass (38–53%) was observed in the BTX-injected hind limb when compared to the contralateral limb. No difference in the magnitude of muscle wasting was found between any of the four mouse strains (Table 1).

The length of the femora was not influenced by BTX in any of the mouse strains (Table 1).

### 3.3 Dual-energy X-ray absorptiometry

Immobilization resulted in significantly lower femoral BMC and aBMD in the BTX-injected hind limb compared with the contralateral limb in the BALB/c and B6 strains, whereas no significant differences were observed in the DBA and C3H strains (Table 1).

The difference in femoral BMC was significantly greater in BALB/c (− 10.9%) and B6 (− 12.9%) compared to C3H (+ 4.2%) mice. Similarly, the loss of femoral aBMD was significantly more pronounced in BALB/c (− 8.8%), B6 (− 10.0%), and DBA (− 5.6%) than in C3H (− 1.0%) mice.

### 3.4 Mechanical testing

**Fig. 1:**
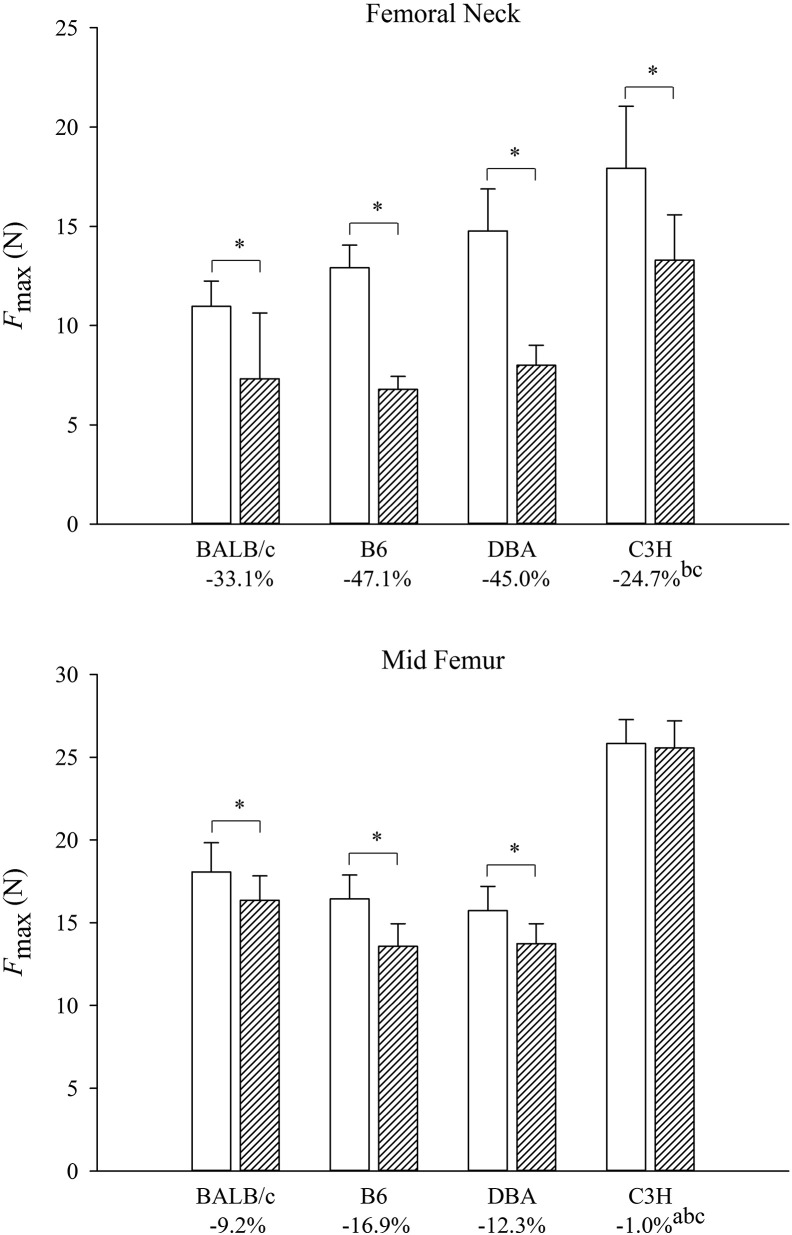
Femoral neck and mid-diaphyseal bone strength. The white columns are the contralateral limbs and the hatched columns are the BTX-injected limbs. Mean differences between limbs are listed in percentages below their respective mice strains. Mean ± SD. *: *p* < 0.05 vs. contralateral limb, a: difference *p* < 0.05 vs. BALB/c, b: difference *p* < 0.05 vs. B6, c: difference *p* < 0.05 vs. DBA.

The bone strength of the femoral neck was significantly lower in the BTX-injected than in the contralateral limb in all mouse strains (Fig. 1). These differences in bone strength were significantly greater in B6 (− 47.1%) and DBA (− 45.0%) than in C3H (− 24.7%) mice.

At the femoral mid-diaphysis, the bone strength was significantly lower in the BTX-injected than in the contralateral limb in BALB/c, B6, and DBA mice, but not in C3H mice. These BTX-induced differences in mid-diaphyseal bone strength was significantly greater in BALB/c (− 9.2%), B6 (− 16.9%), and DBA (− 12.3%) than in C3H (− 1.0%) mice (Fig. 1).

### 3.5 μCT

**Fig. 2:**
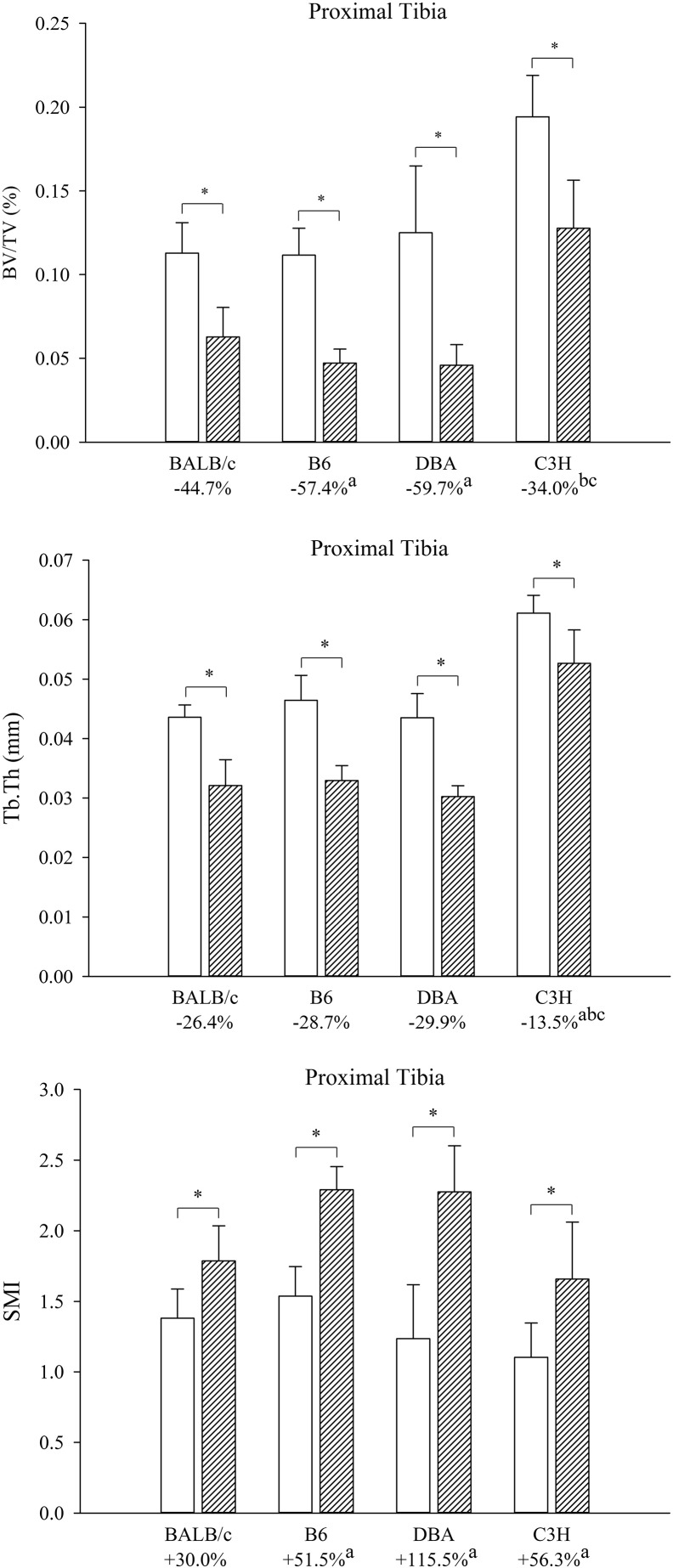
Proximal tibial metaphysis BV/TV, Tb.Th, and SMI. The white columns are the contralateral limbs and the hatched columns are the BTX-injected limbs. Mean differences between limbs are listed in percentages below their respective mice strains. Mean ± SD. *: *p* < 0.05 vs. contralateral limb, a: difference *p* < 0.05 vs. BALB/c, b: difference *p* < 0.05 vs. B6, c: difference *p* < 0.05 vs. DBA.

**Fig. 3:**
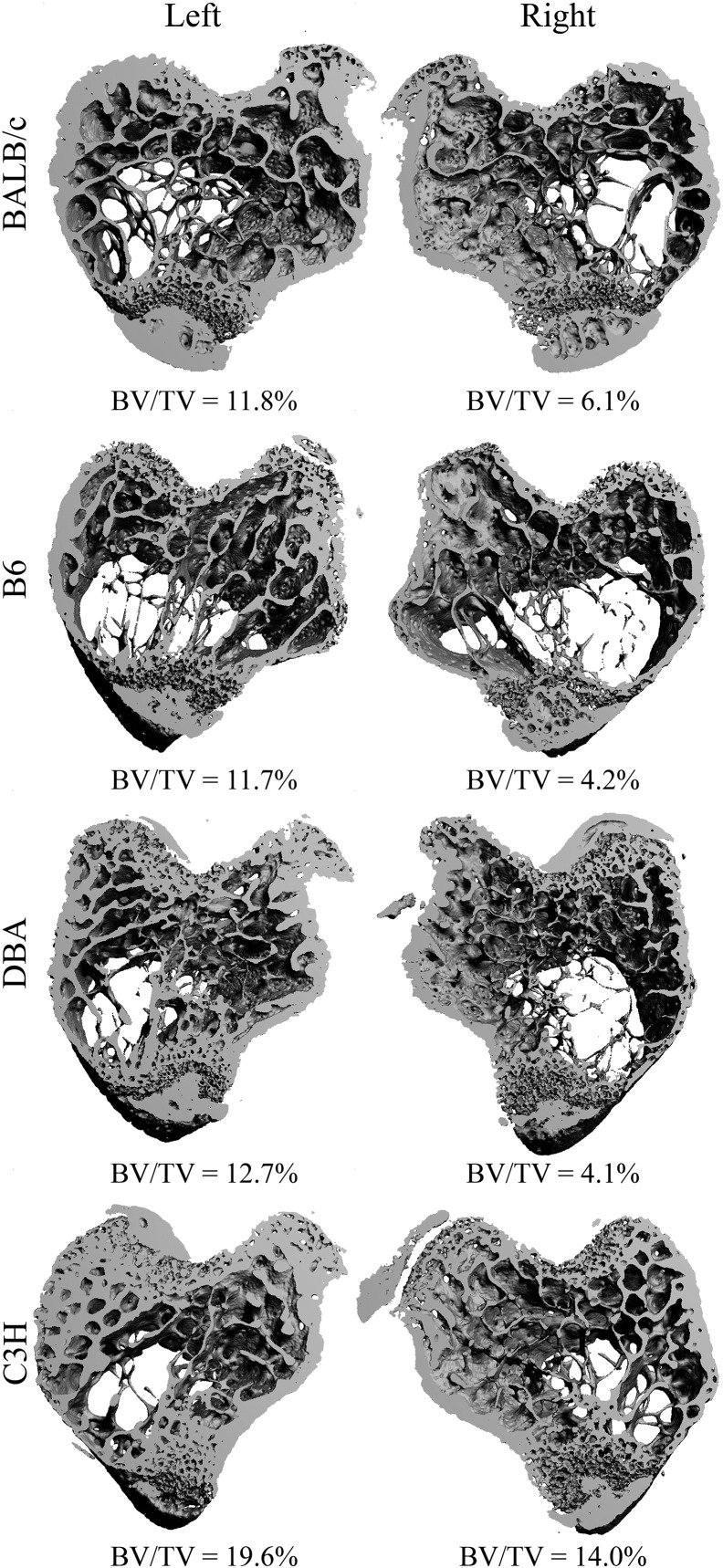
Proximal tibial metaphyses from the BTX group presented as 3D images. Contralateral (left) and BTX-injected (right) limbs are accompanied by their respective BV/TV values.

Compared with the contralateral limb BTX resulted in substantially lower BV/TV values in all mouse strains ranging from − 34% (C3H) to − 60% (DBA). Similarly, the BTX-injections led to significantly lower Tb.Th, CD, vBMD, and ρ_trab_ values and a significantly higher SMI value in all mouse strains (Table 2, Fig. 2, Fig. 3).

The difference in BV/TV was significantly greater in B6 (− 57%) and DBA (− 60%) than in BALB/c (− 45%) and C3H (− 34%) mice. The loss of Tb.Th was significantly greater in BALB/c (− 26.4%), B6 (− 28.7%), and DBA (− 29.9%) than in C3H (− 13.5%) mice. The BTX-induced differences in Tb.N, Tb.Sp, CD, and ρ_trab_ did not differ between the mouse strains. The increase in SMI was significantly greater in B6 (+ 51.5%), DBA (+ 115.5%), and C3H (+ 56.3%) than in BALB/c (+ 30%) mice. Finally, the loss of vBMD was significantly greater in BALB/c (− 58%), B6 (− 80%), and DBA (− 75%) than in C3H (− 36%) mice.

### 3.6 Static histomorphometry of cortical bone

**Fig. 4:**
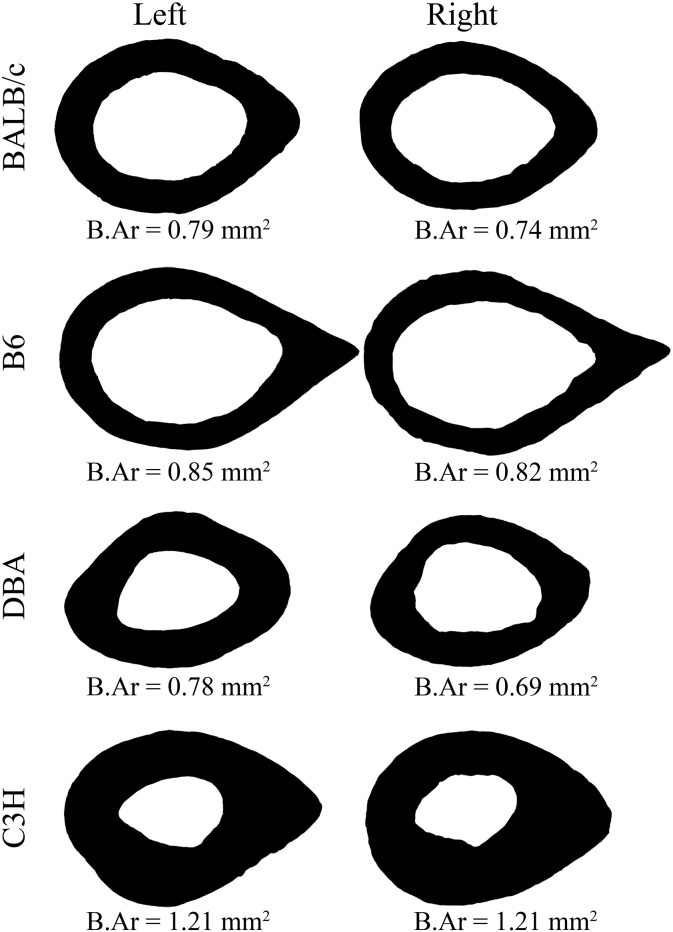
Cross sections of the mid-diaphyseal femur from the BTX group. Contralateral (left) and BTX-injected (right) limbs are accompanied by their respective B.Ar values.

The femoral mid-diaphyseal B.Ar was significantly lower in the BTX-injected limb in B6 and DBA mice compared to the contralateral limb, whereas no difference was found for BALB/c and C3H mice (Table 1 and Fig. 4). These differences were significantly greater in DBA (− 14.6%) than in B6 (− 2.4%) and C3H (+ 0.6%) mice. The M.Ar was significantly larger in the BTX-injected limb in all four mouse strains, but the magnitude of the difference in marrow area did not differ between the mouse strains. Finally, the T.Ar was significantly greater in the BTX-injected limb in the B6 mice, while it proved significantly lower in the BTX-injected limb in DBA mice. This difference in T.Ar between the injected and the contralateral limb was significantly greater in DBA (− 6.0%) than in BALB/c (+ 0.5%), B6 (+ 1.7%), and C3H (+ 3.1%) mice.

### 3.7 Dynamic histomorphometry of cortical bone

At the endocortical surface of the femoral mid-diaphyseal Alz.S/BS was lower in the BTX-injected limb in B6, DBA, and C3H mice, whereas Alz.S/BS was not significantly affected by BTX in BALB/c mice (Table 3).

Endocortical MS/BS was significantly lower in the BTX-injected limbs in BALB/c, B6, and C3H mice, whereas no difference was found in the DBA strain. BTX did not influence MAR and BFR/BS in any of the mouse strains.

No effect of BTX at the periosteal surface was found in any of the mouse strains.

### 3.8 Dynamic histomorphometry of trabecular bone

At the proximal tibia, MS/BS, MAR, BFR/BS, Oc.S/BS, and AV/MV did not differ significantly between the BTX-injected and the contralateral limb in any of the mouse strains (Table 3).

### 3.9 Assessment of the systemic effect of the BTX-injection

In order to address the possibility of BTX having a systemic effect, we compared the contralateral (non-injected) hind limb of the BTX group with the non-injected limb of the control group. BTX did not affect the femoral length or bone strength (both femoral neck and femoral mid-diaphysis were tested) in any mouse strain. Likewise, no differences in rectus femoris muscle mass were found apart from in the C3H mice (− 16%). The aBMD was significantly lower in DBA (− 6%) and C3H (− 5%) mice, but did not differ in the BALB/c and B6 mice. Importantly, none of the microstructural indices differed between the contralateral (non-injected) hind limb in the BTX group and the saline-injected limb in the control group.

## 4 Discussion

The present study established that the response to BTX-induced osteopenia is mouse strain specific. In particular, we found, that even though the loss of muscle mass in C3H mice was comparable to that of B6, DBA, and BALB/c mice, the C3H mice were surprisingly less susceptible to BTX-induced losses of BV/TV, Tb.Th, and bone strength.

In all investigated mouse strains, BTX resulted in loss of BV/TV and Tb.Th accompanied by a decline in femoral bone strength (except for C3H mice). These findings are consistent with previous studies investigating BTX-induced immobilization in both mice and rats, and validate the reproducibility of the BTX-induced immobilization model in mice (Warner et al., 2006a, Warden et al., 2013, Brüel et al., 2013, Ellman et al., 2014, Manske et al., 2010a, Aliprantis et al., 2012, Grimston et al., 2007, Manske et al., 2010b, Manske et al., 2012, Poliachik et al., 2010).

The apparent greater resilience of C3H mice to BTX-induced decreases in BV/TV, Tb.Th, and bone strength was also encountered in previous studies of tail suspended and sciatic neurectomized mice (Kodama et al., 1999, Amblard et al., 2003, Judex et al., 2002, Judex et al., 2004). This relatively greater resistance of C3H mice to immobilization-induced bone loss seems more pronounced in cortical bone than in trabecular bone. In contrast to the other mouse strains, neither aBMD nor femoral mid-diaphyseal bone strength was influenced by BTX in C3H mice. Although immobilization increased the marrow area and decreased endosteal Alz.S/BS in C3H mice, indicating an increased resorption at the endocortical surface, the femoral mid-diaphyseal bone area and tissue area were also increased in these mice indicating a concomitant periosteal drift. This drift may explain the preservation of cortical mechanical strength in immobilized C3H mice.

Interestingly, previous studies of immobilized B6 and C3H mice indicate a surge in osteoclastogenesis and mobilization of osteoclasts as a sizeable contributor to bone loss following unloading (Sakai et al., 2005, Rantakokko et al., 1999, Aliprantis et al., 2012, Poliachik et al., 2010, Amblard et al., 2003, Sakata et al., 1999). Bone marrow from B6 mice has been shown to consistently produce and retain more osteoclasts than bone marrow from C3H mice (Linkhart et al., 1999). The less potent osteoclastogenesis and the lower number of osteoclasts in C3H mice may explain why this mouse strain appears to be less affected by unloading than other strains of mice with greater ‘osteoclastic responsiveness’, notably the B6 strain. Consistent with this, we found that Oc.S/BS was 25% greater (borderline significant) in the BTX-injected than in the contralateral limb in B6 mice, while this difference was only 8% (not significant) in C3H mice. Paradoxically, however, we also found that the contralateral limb of C3H mice had a significantly greater absolute Oc.S/BS compared with the contralateral limb of the B6 strain. In light of these contrasting data our study may, therefore, neither corroborate nor reject the theory that the C3H strain is less affected by immobilization due to inherently less efficient osteoclasts and osteoclastogenesis.

We found no effect of BTX on MS/BS in the trabecular compartment in any mouse strain. This should not be taken as a sign that there was no cellular response from the osteoblasts to the immobilization occurring at all. Instead, the powerful cellular response to unloading is transient and most intense for both osteoblasts and osteoclasts in the early phase of immobilization after which the response to unloading fades out (Sakai et al., 2005, Poliachik et al., 2010, Amblard et al., 2003).

Trabecular bone is characterized by a higher turnover than cortical bone and this may explain why we are still able to find significant BTX-induced differences in MS/BS at the femoral mid-diaphysis at the end of the study.

Mouse strain specific bone loss has previously been shown in the OVX model of bone loss (Bouxsein et al., 2005). However, the mechanisms, leading to bone loss in the OVX model may very likely differ from those of the immobilization models. Bouxsein et al. found that OVX induced significant losses of BV/TV and Tb.Th in C3H and BALB/c mice, whereas, interestingly, the B6 and the DBA strains did not undergo any significant deterioration in trabecular BV/TV and Tb.Th at the proximal tibial metaphysis when compared with sham groups (Bouxsein et al., 2005). Thus, it would appear as if OVX-induced bone loss behave in a different way than BTX-induced bone loss. Here, we found that the C3H mice were significantly less affected by immobilization than B6 and DBA mice.

Furthermore, Bouxsein et al. hypothesized, that mice with an initially low BV/TV cannot afford to lose more bone, and suggested the existence of a possibly new mechano-stat driven feedback system that allows them to maintain trabecular bone volume (Bouxsein et al., 2005). In the present study the mouse strain with the lowest initial BV/TV (B6) was also the mouse strain that was most affected by immobilization, whereas the mouse strain with the highest initial BV/TV (C3H) was actually the mouse strain that was least affected by immobilization. Consequently, our data do not support the notion of a mechano-stat driven feedback system in immobilization models where mice with an initial low BV/TV are protected from further bone loss, but this does not rule out its possible existence in the OVX model.

Skeletal unloading may also be achieved by use of tail suspension. The tail suspension model was initially developed to mimic microgravity conditions during space flight (Morey, 1979). In contrast to the BTX-model, only the ground reaction forces are removed in tail suspended animals, while forces originating from muscle contractions are still present. Several studies of tail suspended mice – comparable to the present study in terms of age and duration of disuse – indicate, that tail suspended BALB/c mice lose BV/TV and Tb.Th in measures roughly equal to that of BTX induced osteopenia (Judex et al., 2002, Judex et al., 2004). Interestingly, this does not seem to be the case for C3H and B6 mice (Warner et al., 2006a, Amblard et al., 2003, Judex et al., 2002, Judex et al., 2004). For most parameters the C3H mice were unaffected in the BTX model, except for BV/TV where we found a significant decrease. However, three comparable studies of tail suspended C3H mice found no differences in BV/TV between control and tail suspended groups (Amblard et al., 2003, Judex et al., 2002, Judex et al., 2004). Thus, BTX-immobilization appears to have a stronger detrimental effect on bone in C3H mice than tail suspension does. In B6 mice, significant losses of BV/TV and Tb.Th occur in both models of immobilization, but the degree of bone deterioration with BTX-immobilization in the present study is between two and three times greater than the losses found with tail suspension (Warner et al., 2006a, Amblard et al., 2003, Judex et al., 2002, Judex et al., 2004). This is corroborated by the findings of Ellman et al. who also found BTX treatment of B6 mice induced two to three times greater bone losses than experienced with tail suspension (BV/TV: − 66% with BTX compared to − 28% with tail suspension) (Ellman et al., 2014). This indicates that BTX results in greater bone losses than tail suspension.

The systemic effect of BTX seems ever present among the studies applying BTX to achieve immobilization and is often mentioned (Warner et al., 2006a, Ellman et al., 2014, Manske et al., 2010a, Grimston et al., 2007, Manske et al., 2010b, Poliachik et al., 2010); but the degree of discrepancy between studies on this matter is perplexing. Most studies find only minor systemic effects (Manske et al., 2010a, Manske et al., 2010b, Poliachik et al., 2010), whereas a study by Ellman et al. has reported profound impact on the contralateral limb (non-injected) compared with control animals (Simpson, 1981).

In the present study, we used a very high spatial resolution (3.5 μm) in our μCT scans and found no evidence of a systemic effect on microstructural parameters such as BV/TV and Tb.Th. Likewise, we found no evidence of a systemic effect of BTX in our measurements of bone strength. However, we did find that the aBMD was 5–6% lower in the non-injected limb compared with the controls in C3H and DBA mice indicating a slight systemic effect of BTX in these mouse strains. A reason for the discrepancy between the large systemic effects of BTX found by Ellman et al. compared with the modest systemic effects found in the present study could be the age of the mice studied. Ellman et al. used 11-week-old B6 mice and mice seemingly experience the least systemic effect of BTX when they are older than 15 weeks (Manske et al., 2010a, Manske et al., 2010b, Poliachik et al., 2010), which may explain the discrepancy.

In conclusion, the present study found that immobilization osteopenia induced by BTX causes severe loss of cortical and trabecular bone and bone strength in B6 and DBA mice. A medium loss of bone microstructure and strength was observed in the BALB/c mice, while the C3H strain was markedly less affected in the microstructural parameters and the femoral neck, and moreover the mid-diaphyseal bone strength was completely unaffected. Importantly, only minimal systemic effects of BTX were found in the present study.

## 5 Disclosures

All authors state that they have no conflicts of interest.

## Figures and Tables

**Table 1:** Body weights of control and BTX groups. For the BTX groups only: rectus femoris muscle mass, femur length, whole femur DXA values, and static histomorphometry of the femoral mid-diaphysis.

	BALB/c		B6		DBA		C3H	
	Start	End	Start	End	Start	End	Start	End
*Body weight*								
Control group (g)	20.5 (± 1.4)	22.1# (± 1.2)	21.4 (± 1.2)	21.8 (± 1.5)	22.3 (± 0.7)	22.5 (± 0.7)	25.0 (± 1.5)	26.7# (± 1.2)
BTX group (g)	21.5 (± 1.5)	20.3#, ǂ (± 1.3)	22.2 (± 0.9)	21.3# (± 1.1)	22.1 (± 0.7)	20.9#, ǂ (± 0.7)	25.1 (± 1.3)	23.4#, ǂ (± 1.2)

BTX group	Contralat.	Inj.	Contralat.	Inj.	Contralat.	Inj.	Contralat.	Inj.

M. rectus femoris (mg)	56.0 (± 5.1)	34.3⁎ (± 5.3)	62.3 (± 3.6)	33.4⁎ (± 7.4)	62.5 (± 13.6)	29.4⁎ (± 2.6)	56.5 (± 3.9)	30.2⁎ (± 7.7)
	− 38.3% (± 11.4)		− 46.2% (± 12.2)		− 52.5% (± 8.2)		− 46.3% (± 14.1)	
Femur length (mm)	15.03 (± 0.3)	15.06 (± 0.4)	15.40 (± 0.3)	15.38 (± 0.2)	14.93 (± 0.1)	14.88 (± 0.1)	15.61 (± 0.2)	15.59 (± 0.2)
	0.2% (± 1.1)		− 0.1% (± 0.9)		− 0.3% (± 0.8)		− 0.2% (± 1.1)	
*Whole femur*								
BMC (mg)	17.8 (± 2.3)	15.6⁎ (± 1.3)	18.1 (± 1.0)	15.7⁎ (± 1.3)	15.1 (± 1.1)	14.2 (± 1.5)	25.1 (± 2.8)	25.8 (± 2.8)
	− 10.9% (± 14.5)		− 12.9% (± 9.7)		− 5.6% (± 11.2)		4.2%^a^, ^b^ (± 17.6)	
aBMD (mg/cm^2^)	55.6 (± 3.0)	50.7⁎ (± 2.8)	51.7 (± 1.9)	46.5⁎ (± 1.9)	51.0 (± 3.2)	47.9 (± 1.6)	74.8 (± 3.0)	74.0 (± 3.2)
	− 8.8% (± 4.1)		− 10.0% (± 5.7)		− 5.6% (± 8.3)		− 1.0%^a^, ^b^, ^c^ (± 3.5)	

*Femoral mid-diaphysis*								
Bone Area (mm^2^)	0.81 (± 0.08)	0.78 (± 0.07)	0.85 (± 0.06)	0.83⁎ (± 0.07)	0.79 (± 0.04)	0.77⁎ (± 0.25)	1.23 (± 0.06)	1.24 (± 0.07)
	− 3.8% (± 11.5)		− 2.4% (± 2.5)		− 14.6%^b^ (± 5.9)		0.6%^c^ (± 4.4)	
Marrow area (mm^2^)	0.63 (± 0.06)	0.67⁎ (± 0.07)	0.91 (± 0.05)	0.96⁎ (± 0.04)	0.41 (± 0.01)	0.44⁎ (± 0.03)	0.34 (± 0.07)	0.38⁎ (± 0.06)
	6.5% (± 6.4)		5.6% (± 3.4)		10.8% (± 5.7)		14.0% (± 17.0)	
Tissue area (mm^2^)	1.44 (± 0.12)	1.44 (± 0.12)	1.75 (± 0.08)	1.78⁎ (± 0.09)	1.20 (± 0.04)	1.13⁎ (± 0.05)	1.58 (± 0.10)	1.62 (± 0.10)
	0.5% (± 7.7)		1.7% (± 1.3)		− 6.0%^a^, ^b^ (± 4.0)		3.1%^c^ (± 4.7)	

Mean differences are listed in percentage between the BTX-injected and contralateral limbs.

Mean ± SD.

# *p* < 0.05 vs. start weight.

ǂ *p* < 0.05 vs. control group.

⁎ *p* < 0.05 vs. contralateral limb.

a Difference *p* < 0.05 vs. BALB/c.

b Difference *p* < 0.05 vs. B6,

c difference *p* < 0.05 vs. DBA.

**Table 2:** Trabecular bone micro-structure indices of the proximal tibial metaphysis.

	BALB/c		B6		DBA		C3H	
	Contralat	Inj.	Contralat	Inj.	Contralat	Inj.	Contralat	Inj.
*Tibial proximal metaphysis*								
BV/TV (%)	11.3 (± 1.8)	6.3⁎ (± 1.7)	11.2 (± 1.6)	4.7⁎ (± 0.8)	12.5 (± 4.0)	4.6⁎ (± 1.2)	19.4 (± 2.5)	12.8⁎ (± 2.9)
	− 45% (± 10)		− 57%^a^ (± 8)		− 60%^a^ (± 18)		− 34%^b^, ^c^ (± 13)	
Tb.Th (μm)	44 (± 2)	32⁎ (± 4)	46 (± 4)	33⁎ (± 3)	44 (± 4)	30⁎ (± 2)	61 (± 3)	53⁎ (± 6)
	− 26.4% (± 9.1)		− 28.7% (± 6.2)		− 29.9% (± 8.7)		− 13.5%^a^, ^b^, ^c^ (± 10.7)	
Tb.N (mm^− 1^)	3.66 (± 0.30)	3.37⁎ (± 0.22)	3.69 (± 0.24)	3.39⁎ (± 0.16)	3.67 (± 0.43)	3.25⁎ (± 0.31)	3.86 (± 0.41)	3.46 (± 0.40)
	− 7.7% (± 5.9)		− 7.8% (± 6.0)		− 10.7% (± 9.7)		− 9.6% (± 14.4)	
Tb.Sp (μm)	274 (± 25)	294⁎ (± 21)	268 (± 19)	291⁎ (± 15)	284 (± 30)	310⁎ (± 32)	266 (± 34)	292 (± 36)
	7.8% (± 7.3)		8.7% (± 7.3)		10.1% (± 12.2)		11.2% (± 16.0)	
CD (mm^3^)	197 (± 48)	120⁎ (± 39)	186 (± 38)	98⁎ (± 39)	309 (± 121)	151⁎ (± 68)	235 (± 45)	165⁎ (± 59)
	− 39.0% (± 12.9)		− 47.5% (± 15.5)		− 43.1% (± 37.3)		− 26.9% (± 30.4)	
SMI (−)	1.4 (± 0.2)	1.8⁎ (± 0.2)	1.5 (± 0.2)	2.3⁎ (± 0.2)	1.2 (± 0.4)	2.3⁎ (± 0.3)	1.1 (± 0.2)	1.7⁎ (± 0.4)
	30.0% (± 10.4)		51.5%^a^ (± 22.7)		115.5%^a^ (± 120.4)		56.3%^a^ (± 52.0)	
vBMD (mg/cm^3^)	113 (± 25)	48⁎ (± 24)	105 (± 18)	22⁎ (± 25)	120 (± 43)	26⁎ (± 18)	208 (± 39)	129⁎ (± 35)
	− 58% (± 16)		− 80% (± 25)		− 75% (± 21)		− 36%^a^, ^b^, ^c^ (± 19)	
ρ_trab_	971 (± 25)	915⁎ (± 28)	906 (± 22)	852⁎ (± 20)	917 (± 25)	856⁎ (± 22)	1019 (± 11)	991⁎ (± 27)
	− 5.7% (± 3.7)		− 5.9% (± 3.2)		− 6.6% (± 4.5)		− 2.7% (± 2.5)	

Mean differences are listed in percentage between the BTX-injected and contralateral limbs.

Mean ± SD.

⁎ *p* < 0.05 vs. contralateral limb.

a Difference *p* < 0.05 vs. BALB/c.

b Difference *p* < 0.05 vs. B6.

c Difference *p* < 0.05 vs. DBA.

**Table 3:** Dynamic histomorphometry of the mid-diaphyseal femur (cortical) and the proximal tibial metaphysis (trabecular).

	BALB/c		B6		DBA		C3H	
	Contralat	Inj.	Contralat	Inj.	Contralat	Inj.	Contralat	Inj.
*Femoral mid-diaphysis*								
e.Alz/BS (−)	0.18 (± 0.16)	0.16 (± 0.16)	0.50 (± 0.13)	0.28⁎ (± 0.12)	0.44 (± 0.15)	0.20⁎ (± 0.12)	0.41 (± 0.13)	0.19⁎ (± 0.10)
	− 54.6% (± 52.9)		− 40.1% (± 21.9)		− 57.8% (± 24.9)		− 46.8% (± 38.1)	
e.MS/BS (−)	0.12 (± 0.08)	0.03⁎ (± 0.04)	0.30 (± 0.19)	0.16⁎ (± 0.15)	0.14 (± 0.07)	0.13 (± 0.12)	0.23 (± 0.09)	0.14⁎ (± 0.07)
	− 64.1% (± 65.7)		− 43.1% (± 58.5)		− 3.9% (± 124.1)		− 34.7% (± 36.7)	
p.Alz/BS (−)	0.75 (± 0.22)	0.68 (± 0.27)	0.59 (± 0.14)	0.57 (± 0.08)	0.70 (± 0.16)	0.72 (± 0.19)	0.79 (± 0.14)	0.92 (± 0.07)
	− 2.1% (± 50.4)		1.1% (± 29.7)		0.0% (± 21.4)		15.7% (± 27.6)	
p.MS/BS (−)	0.18 (± 0.07)	0.20 (± 0.05)	0.18 (± 0.12)	0.21 (± 0.07)	0.28 (± 0.11)	0.18 (± 0.09)	0.23 (± 0.15)	0.36 (± 0.13)
	28.8% (± 76.9)		36.8% (± 78.4)		− 34.2% (± 28.3)		52.8% (± 64.8)	

*Tibial proximal metaphysis*								
MS/BS (−)	0.35 (± 0.04)	0.38 (± 0.05)	0.38 (± 0.14)	0.37 (± 0.13)	0.35 (± 0.09)	0.29 (± 0.10)	0.38 (± 0.09)	0.36 (± 0.07)
	9.2% (± 25.9)		− 0.3% (± 20.4)		− 17.2% (± 25.6)		− 1.6% (± 18.3)	
Oc.S/BS (−)	25.95 (± 4.88)	26.55 (± 6.04)	22.67 (± 3.89)	27.68 (± 4.84)	24.34 (± 3.58)	26.19 (± 4.31)	29.79 (± 3.70)	32.08 (± 5.41)
	0.8% (± 27.0)		25.0% (± 26.4)		9.3% (± 27.5)		7.9% (± 18.1)	
AV/MV (%)	0.26 (± 0.49)	0.23 (± 0.21)	1.34 (± 0.85)	1.88 (± 1.35)	1.91 (± 1.08)	1.63 (± 0.98)	1.93 (± 1.76)	1.88 (± 0.87)
	153.4% (± 254.5)		87.9% (± 234.9)		109.1% (± 258.1)		17.7% (± 70.8)	

Mean differences are listed in percentage between the BTX-injected and contralateral limbs.

Mean ± SD.

⁎ *p* < 0.05 vs. contralateral limb.
